# Sepsis Due to Capnocytophaga canimorsus Following a Fox Bite: A Case Report

**DOI:** 10.7759/cureus.107479

**Published:** 2026-04-21

**Authors:** Christelle Rahme, Zara Chaudhry, Denise Mourad, Palaniandy Kogulan, Nicholas Haddad

**Affiliations:** 1 Internal Medicine, Central Michigan University College of Medicine, Mount Pleasant, USA; 2 Internal Medicine, Central Michigan University College of Medicine, Saginaw, USA; 3 Infectious Diseases, Covenant HealthCare, Saginaw, USA; 4 Infectious Diseases/Internal Medicine, Central Michigan University College of Medicine, Saginaw, USA

**Keywords:** animal bite, capnocytophaga canimorsus, case report, culture, fox bite, sepsis, zoonotic infection

## Abstract

*Capnocytophaga canimorsus* is a commensal bacterium found in the oral flora of dogs and cats. Close animal contact with humans through biting, scratching, or wound licking may lead to transmission of this microorganism. Although infections are uncommon, they can be severe, particularly in individuals with predisposing risk factors. Transmission has most commonly been associated with dogs and, less frequently, cats. Reports involving other animal sources remain rare.

We report the case of a male patient who presented to the Emergency Department with fever and abdominal pain approximately one week after sustaining a skin laceration on his right hand from a bite by a domesticated fox. He was promptly started on broad-spectrum antibiotics and responded quickly to treatment. Ten days after discharge from the hospital, blood cultures were reported positive for *C. canimorsus*. Severe infections with this bacterium have been reported in immunocompromised individuals but may also occur in previously healthy persons. Mortality rates from *C. canimorsus* sepsis range between 10% and 30%; therefore, prompt recognition and treatment are essential for favorable outcomes.

Although a definitive causal relationship cannot be established without microbiologic testing of the animal source, the wound the patient presented with, and the recent exposure to a fox, raised concern for this. The temporal association between the fox bite and the patient’s illness raises the possibility of zoonotic transmission. This case highlights the importance of obtaining a detailed exposure history in patients presenting with sepsis of unclear origin and suggests that additional animal reservoirs for *C. canimorsus* may exist.

## Introduction

*Capnocytophaga canimorsus* is a zoonotic pathogen commonly present in the oral flora of dogs and cats. It is a Gram-negative rod that is medium to long in size and often demonstrates a tapered or spindle-shaped morphology [1]. The estimated incidence of infection with this bacterium is approximately 0.5-0.7 cases per million individuals per year [2].

The most frequent clinical presentation is sepsis; however, infections may also manifest as meningitis, endocarditis, mycotic aneurysm, brain abscess, or endophthalmitis [3]. Common presenting symptoms include fever, diarrhea, abdominal pain, vomiting, headache, confusion, and myalgia or malaise. In hospitalized patients, severe complications such as disseminated intravascular coagulation (DIC) or septic shock have been reported [1].

Infection can occur in both immunocompetent and immunocompromised individuals [4]. Patients with underlying conditions such as malignancy, diabetes mellitus, human immunodeficiency virus infection, alcohol use disorder, or functional or anatomical asplenia are at higher risk for developing severe disease [5]. Mortality from severe sepsis secondary to *C. canimorsus* has been reported to reach 26% [6].

The Centers for Disease Control and Prevention (CDC) recommends empiric treatment with a beta-lactam/beta-lactamase inhibitor combination (such as amoxicillin-clavulanic acid). Third-generation cephalosporins (such as ceftriaxone) or carbapenems may also be used. Clindamycin is an alternative in penicillin-allergic patients, often combined with a fluoroquinolone or trimethoprim-sulfamethoxazole. Early empiric therapy is crucial while diagnostic testing is pending, as *C. canimorsus* requires up to 14 days of incubation for culture growth [7].

Transmission to humans most commonly occurs through dog or cat bites, scratches, or licking of open wounds [2]. The organism is typically a commensal inhabitant of the saliva of healthy dogs and cats [8]. Although transmission has predominantly been associated with these animals, rare reports suggest that other carnivorous species may also serve as potential reservoirs. For example, a case of *C. canimorsus* endocarditis following a lion bite has been reported, suggesting possible transmission from additional animal species.

We present a case of *C. canimorsus* bacteremia in a patient who developed sepsis after a bite from a domesticated fox. While microbiologic confirmation from the animal was not obtained, the temporal relationship raises the possibility of zoonotic transmission from this exposure.

## Case presentation

This patient is a male in his early 50s with a past medical history of degenerative joint disease who sustained a superficial wound secondary to a bite from a domesticated fox on his right thumb seven days earlier. Following the bite, he washed the wound and applied hydrogen peroxide. He presented to the Emergency Department with right upper quadrant abdominal pain, nausea, vomiting, and fever. His symptoms began two days prior to presentation. He also reported nasal congestion, sore throat, and two episodes of loose stools. Several family members had recently experienced upper respiratory tract infections, and he initially attributed his symptoms to a viral illness.

**Figure 1:**
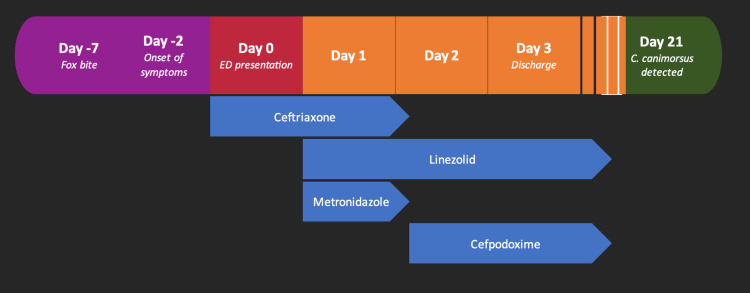
Timeline of Patient's Hospital Course: From Fox Bite to Discharge and Post-discharge Positive Culture Results Clinical timeline and medication course of the patient during their hospital visit. C. canimorsus: Capnocytophaga canimorsus

Upon presentation, the patient was febrile, with a temperature of 39.4°C (102.9°F), tachycardic, with a heart rate of 126 beats/min, and had a respiratory rate of 20 breaths/min. His blood pressure was 118/57 mmHg, and oxygen saturation was 96% on room air. Physical examination revealed an ill-appearing man with dry mucous membranes. Abdominal examination demonstrated generalized tenderness without peritoneal signs. Examination of the right thumb revealed superficial scratches with a scabbed area on the palmar surface, without surrounding erythema, warmth, or purulent drainage (Figure 1).

Initial laboratory investigations demonstrated leukocytosis with neutrophil predominance and markedly elevated inflammatory markers. Mild renal dysfunction was also present, while lactate, liver function tests, and lipase levels remained within normal limits (Table 1).

**Table 1:** Initial Laboratory Values Obtained During the Emergency Department Visit

Laboratory Test	Patient Value	Reference Range	Interpretation
White blood cell count	24.83 × 10⁹/L	3.4-11 × 10⁹/L	Increased
Neutrophils	94.5%	40%-70%	Increased
C-reactive protein	182 mg/L	<7.5 mg/L	Increased
Procalcitonin	26 ng/mL	0-0.10 ng/mL	Increased
Creatinine	1.1 mg/dL	0.6-1.0 mg/dL	Mildly increased

**Figure 2:**
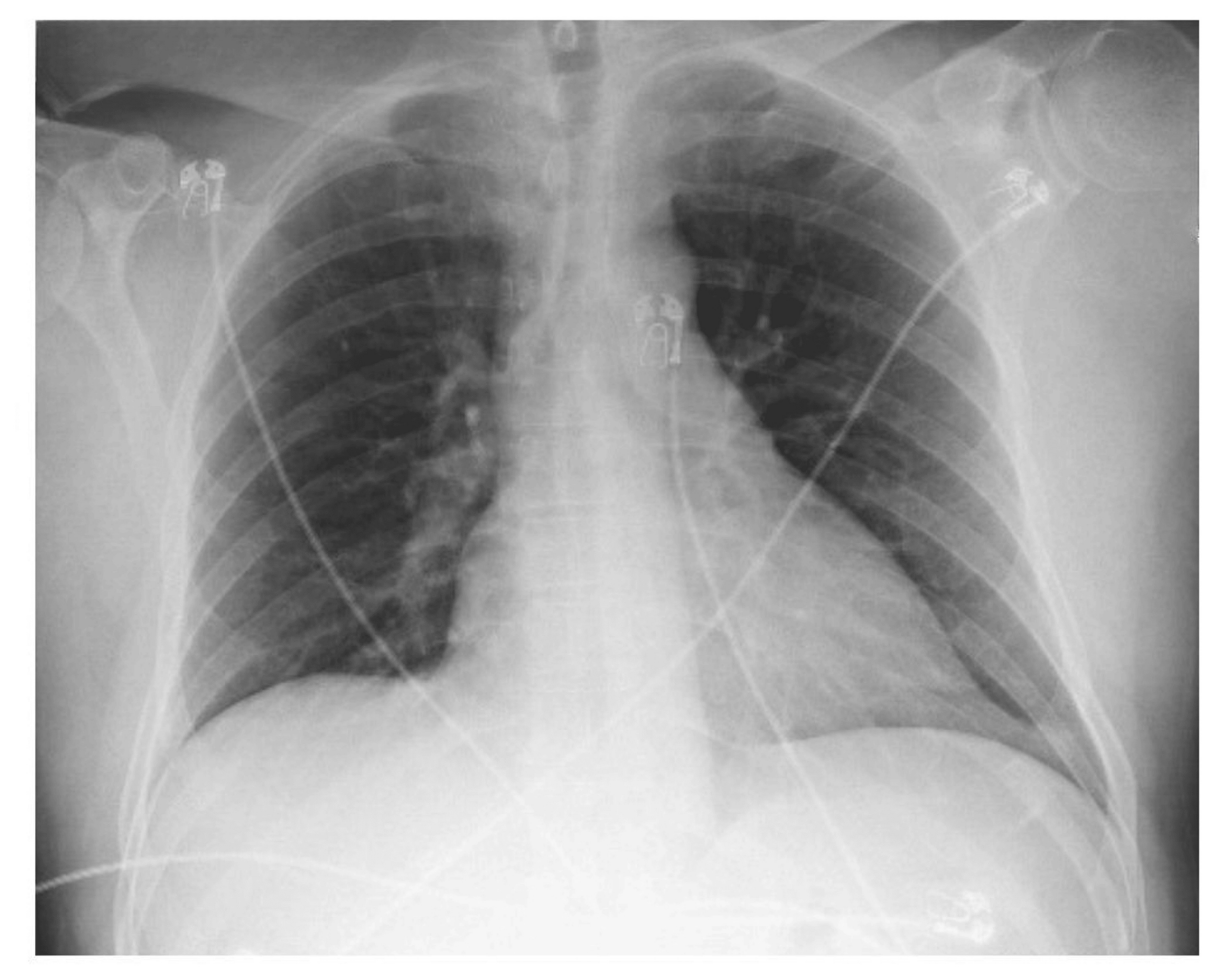
Chest Radiograph Posteroanterior chest radiograph demonstrates a normal cardiomediastinal silhouette. Pulmonary vasculature is normal in caliber and distribution. The lungs are clear bilaterally, with no evidence of consolidation, infiltrates, or masses. No pleural effusions are identified. The costophrenic angles are clear. No pneumothorax is present. Osseous structures are unremarkable.

**Figure 3:**
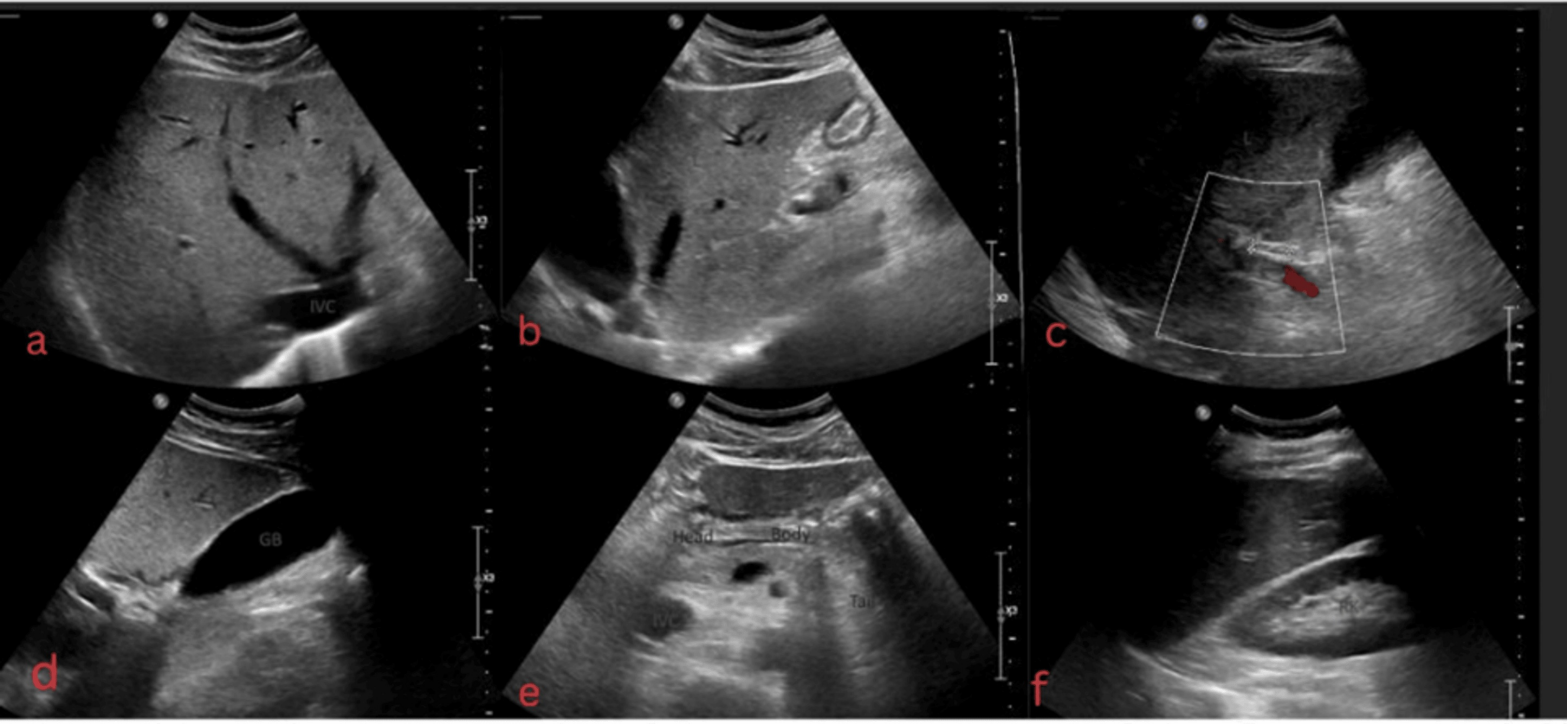
Transabdominal Ultrasound of the Abdomen (a) Transverse sonographic image of the right liver and (b) longitudinal ultrasound of the left hepatic lobe demonstrate normal homogeneous parenchyma with increased echogenicity, likely indicating underlying hepatocellular disease. (c) Common hepatic duct and common bile duct are not dilated (measured diameter: 0.464 cm). (d) Longitudinal gallbladder in supine position shows a normal-sized gallbladder; no gallstones are seen, and gallbladder wall thickness is normal. (e) The visualized portion of the pancreas is unremarkable, with normal parenchymal echotexture. (f) Longitudinal image of the right kidney demonstrates normal renal size, cortical thickness, and corticomedullary differentiation. No hydronephrosis, calculi, or focal masses are identified.

A respiratory viral panel, including influenza A and B, respiratory syncytial virus, and SARS-CoV-2, was negative. Chest radiography demonstrated no acute cardiopulmonary abnormalities (Figure 2). Abdominal ultrasound and computed tomography imaging of the abdomen and pelvis did not reveal an identifiable source of infection (Figure 3).

Given that the patient met systemic inflammatory response syndrome (SIRS) criteria with an unclear infectious source, he was started empirically on 2 g of intravenous ceftriaxone and admitted for further evaluation and management after blood cultures were obtained.

The infectious diseases consultation service evaluated the patient the following day and recommended the addition of 600 mg intravenous linezolid to broaden Gram-positive coverage, including methicillin-resistant *Staphylococcus aureus*, given the patient’s systemic inflammatory presentation and unclear infectious source. Metronidazole at 500 mg was subsequently added to expand anaerobic coverage while intra-abdominal sources of infection were being considered.

After two days of antimicrobial therapy, the patient’s fever resolved, and his abdominal symptoms improved. His white blood cell count and inflammatory markers began trending downward. Initial blood and urine cultures remained negative after two days of incubation. Given the patient’s clinical improvement, antimicrobial therapy was transitioned to oral cefpodoxime and linezolid.

During further history-taking, the infectious disease team specifically inquired about animal exposures and zoonotic risk factors, at which point the patient disclosed the recent fox bite that had occurred approximately one week prior to presentation. Given the possibility of zoonotic infection, repeat blood cultures were obtained with consideration for fastidious organisms. The patient continued to improve clinically and was discharged home after a four-day hospitalization, with instructions to complete a seven-day course of 200 mg oral cefpodoxime and 600 mg linezolid for 19 days.

Given the history of an animal bite, tetanus immunization status was reviewed and addressed according to current vaccination guidelines. Rabies exposure risk was also assessed. The patient was instructed to observe the fox for any abnormal behavior for 10 days in accordance with public health recommendations. The animal remained asymptomatic during the observation period, and rabies post-exposure prophylaxis was not initiated.

Three days after discharge, the blood cultures demonstrated anaerobic Gram-negative rods that were not viable for definitive identification in the hospital laboratory and were therefore sent to an outside reference laboratory. Using 16S rRNA gene sequencing, the organism was subsequently identified as *C. canimorsus* approximately three weeks after the initial culture collection (Figure 1). At one-month follow-up, the patient reported complete clinical recovery.

## Discussion

*C. canimorsus* is a facultative anaerobic, capnophilic, Gram-negative rod that typically demonstrates fusiform or filamentous morphology [9,10]. Identification of this organism can be challenging because of its slow growth, often requiring two to seven days of incubation on blood or chocolate agar in a carbon dioxide-enriched environment [11]. Additionally, the organism requires high concentrations of exogenous iron for optimal growth [10].

*C. canimorsus* can evade host immune defenses through resistance to phagocytosis and intracellular killing. Diagnosis is most commonly established through identification of the organism in blood cultures, which occurs in approximately 88% of reported cases [1]. Although Gram staining may provide preliminary information, definitive identification is often achieved using modern techniques such as matrix-assisted laser desorption ionization-time of flight mass spectrometry (MALDI-TOF MS) or molecular methods, including 16S rRNA gene sequencing [10].

Most reported cases of *C. canimorsus* infection involve exposure to dogs, while transmission from cats accounts for fewer than 10% of cases [2]. However, additional animal reservoirs may exist. For instance, a case of *C. canimorsus* endocarditis following a lion bite has been described in the literature, suggesting that transmission from other carnivorous species may occur. Although microbiologic testing of the fox was not performed in our case, the temporal association between the bite and onset of sepsis raises the possibility that foxes could serve as a potential reservoir.

The incubation period for *C. canimorsus* infection in humans typically ranges from 5 to 14 days [2,10]. Early manifestations may include a local lesion at the bite site, followed by cellulitis characterized by erythema, pain, and possible purulent discharge. Regional lymphangitis and lymphadenopathy may develop. Systemic symptoms often resemble an influenza-like illness, including fever, chills, myalgias, and malaise. Gastrointestinal symptoms such as abdominal pain, vomiting, and diarrhea occur in approximately 25%-30% of cases, as observed in our patient [2].

Antimicrobial susceptibility patterns of *C. canimorsus* generally demonstrate sensitivity to penicillins, third-generation cephalosporins, carbapenems, fluoroquinolones, clindamycin, doxycycline, metronidazole, and chloramphenicol [11]. Risk factors for severe infection include alcohol use disorder, immunosuppression, and functional or anatomical asplenia. Patients without these risk factors can still develop severe infection, although this occurs less frequently. Our patient had no known history of splenectomy or functional asplenia, illustrating that severe disease may also occur in otherwise healthy individuals.

Importantly, *Capnocytophaga* species exhibit intrinsic resistance to aminoglycosides, and these agents should not be relied upon for treatment. Beta-lactam antibiotics remain the preferred first-line therapy for severe infections caused by *C. canimorsus* [2,12].

These factors highlight the diagnostic challenges associated with this infection and explain the delayed identification in our case. Prompt recognition and appropriate antimicrobial therapy are essential for improving outcomes. This case also underscores the importance of obtaining a detailed exposure history in patients presenting with sepsis of unclear origin, as identification of animal contact may significantly influence diagnostic considerations and management.

## Conclusions

*C. canimorsus* infection is rare and often difficult to diagnose because of nonspecific symptoms and the organism’s slow growth in culture. A comprehensive clinical history, with particular attention to animal exposures, is essential for raising clinical suspicion and guiding appropriate diagnostic testing and treatment.

Although dogs and cats remain the most recognized reservoirs of this organism, this case highlights the possibility that other animal species may also serve as sources of infection. Clinicians should maintain a high index of suspicion for zoonotic pathogens in patients presenting with sepsis following animal exposure. Further investigation into potential reservoirs of *C. canimorsus* in animals beyond dogs and cats may help clarify additional sources of transmission.
